# Accuracy of large language models in the Turkish dental specialization examination (DUS): a multidimensional evaluation across disciplines and question formats

**DOI:** 10.1186/s12903-026-09758-6

**Published:** 2026-09-01

**Authors:** Fatih Karaaslan, Muhammed Halil Yilan, Merve Tirimoğulları

**Affiliations:** Department of Periodontology, Faculty of Dentistry, Uşak University, Uşak, 64000 Türkiye

**Keywords:** Artificial Intelligence, Large Language Models, Dental Education, Dental Specialization Examination, DUS, Multimodal Assessment, Natural Language Processing

## Abstract

**Background:**

This study aimed to evaluate the performance of contemporary Large Language Models (LLMs) on the clinical sciences component of the Turkish Dental Specialization Examination (DUS) by comparing their accuracy across disciplines, examination years, and question types.

**Methods:**

The source dataset contained 1,040 scheduled clinical-science questions from the official DUS examinations (2012/1–2021). Thirteen officially cancelled items were excluded, leaving 1,027 evaluable questions across eight disciplines (871 MCQs, 119 CMCQs, and 37 IBMCQs). The same questions were submitted once to seven consumer AI interfaces. Overall and exploratory subgroup comparisons were performed using Cochran’s Q test. Only the overall comparison was followed by 21 exact pairwise McNemar tests with Holm adjustment. Wilson 95% confidence intervals were calculated for overall accuracy.

**Results:**

Overall accuracy differed among the seven interfaces (Cochran’s Q = 669.10; *p* < 0.001). Gemini 2.5 Pro achieved the highest accuracy (92.9%; 954/1,027), whereas Qwen3-Max achieved the lowest (59.5%; 611/1,027). Exploratory Cochran’s Q tests indicated omnibus differences among interfaces within every dental discipline (all *p* < 0.001) and within MCQ (Q = 669.91; *p* < 0.001), CMCQ (Q = 59.29; *p* < 0.001), and IBMCQ groups (Q = 14.68; *p* = 0.023). The IBMCQ result should be interpreted cautiously because only 37 items were available.

**Conclusions:**

Under standardized, single-attempt Turkish DUS testing conditions, the evaluated consumer AI interfaces showed materially different accuracy across the full dataset and exploratory discipline- and format-based subgroups. These findings characterize examination performance only and require confirmation with novel, unpublished, clinically contextualized, and independently assessed questions before broader educational or clinical utility can be inferred.

**Supplementary Information:**

The online version contains supplementary material available at https://doi.org/10.1186/s12903-026-09758-6.

## Background

Large Language Models (LLMs) are advanced artificial intelligence (AI) systems capable of understanding and generating human language through deep learning and large-scale neural network architectures. Trained on extensive textual corpora that include books, scientific literature, and online resources, LLMs have been studied across healthcare for scientific writing, patient education, multilingual communication, health professions education, and the retrieval and synthesis of clinical information [1, 2]. Recent studies in medicine and dentistry suggest that LLMs may enhance learning efficiency and provide meaningful support in complex decision-making scenarios [3, 4].

Dentistry represents a particularly challenging field for AI-driven reasoning due to its reliance on visual–spatial interpretation, specialty-specific biomechanical principles, and the integration of radiographic findings with contemporary clinical guidelines. Questions in specialties such as periodontology, oral and maxillofacial surgery, orthodontics, and prosthodontics may require both knowledge recall and the integration of clinical, radiographic, and biomechanical information [5, 6]. Consequently, evaluating LLM performance solely through overall accuracy is insufficient; performance must be examined across multiple dimensions, including discipline-specific variability, question format, and cognitive complexity.

In Türkiye, entry into postgraduate specialty programs has been regulated since 2012 through the Dental Specialization Examination (DUS), administered by the Assessment, Selection, and Placement Center (ÖSYM). The DUS is grounded in internationally recognized dental textbooks and encompasses eight clinical dental specialties, assessing both theoretical knowledge and applied clinical reasoning [7–9]. These characteristics render the DUS a robust platform for evaluating LLM performance within a standardized and comprehensive dental assessment framework.

Emerging research indicates that LLM accuracy varies widely depending on a model’s architecture, training data, and the modality of the presented questions [2, 10]. Standard multiple-choice questions (MCQs) primarily measure factual recall, whereas complex multiple-choice questions (CMCQs), which require evaluating multiple statements simultaneously, engage integrative reasoning processes [2, 6]. Image-based multiple-choice questions (IBMCQs), incorporating radiographs and clinical photographs, have been shown to be especially challenging for current AI systems, reflecting ongoing limitations in multimodal understanding [11].

Although recent studies have explored LLM accuracy in selected dental specialties [7, 12], there remains a lack of comprehensive analyses that incorporate official DUS clinical sciences questions from multiple years, various question types, and multiple contemporary LLMs. This gap limits our understanding of specialty-specific performance, question-type sensitivity, and year-based stability. To address these gaps, the present study evaluates DUS clinical sciences questions from the 2012/1–2021 examination periods across seven contemporary AI models. Prior studies have generally been limited in scope: most evaluated a single model or a small number of models [1, 4], focused on a single dental specialty or specialty-oriented dental task [13–19], did not examine question-format effects [6, 7], used narrower specialty, examination, language, or educational settings [20–26], or used non-Turkish materials [3, 4, 12]. To our knowledge, based on the literature available up to January 2026, no previous study has combined evaluation of seven LLMs across all eight DUS clinical dental specialties, three question formats (MCQ, CMCQ, IBMCQ), and a ten-year examination period within a single standardized Turkish-language dataset. The study focuses on the following research questions:

(i) Do consumer AI interfaces exhibit significant accuracy differences across dental specialties?
(ii) Does interface performance vary across examination-period question sets?
(iii) Do consumer AI interfaces demonstrate significant accuracy differences across MCQ, CMCQ, and IBMCQ formats?
(iv) How do discipline × question-format strata influence interface accuracy?

By addressing these questions, this study aims to provide a comprehensive and evidence-based assessment of the capabilities and limitations of current LLMs in dentistry, informing their cautious use in dental education, examination preparation, and AI-assisted learning environments.

## Methods

This was a cross-sectional comparative evaluation study. This study was conducted in accordance with the Declaration of Helsinki. Since only publicly available and official examination materials were used, no human participants or animal subjects were involved; therefore, ethical approval was not required. This methodological approach aligns with previous research evaluating LLMs using standardized examination datasets [2, 10].

### Dataset

The source dataset consisted of 1,040 scheduled clinical-science questions from DUS examinations administered by ÖSYM between 2012/1 and 2021. Thirteen questions officially cancelled by ÖSYM were excluded before analysis, leaving 1,027 evaluable questions. The study used a census of all eligible clinical-science questions in the prespecified period rather than a sampled subset; therefore, no a priori power analysis or sample-size calculation was performed. Including the complete eligible question population partially compensates for the absence of an a priori calculation because no subset had to be selected from the defined study frame. It does not, however, overcome the limited precision of small subgroups such as IBMCQs.

Questions were categorized into eight core dental clinical disciplines represented in the DUS:

1. Restorative Dentistry
2. Prosthodontics
3. Oral and Maxillofacial Surgery
4. Oral and Maxillofacial Radiology
5. Periodontology
6. Orthodontics
7. Endodontics
8. Pediatric Dentistry

To evaluate reasoning complexity, each question was further classified into one of three structural formats:

- MCQ (Multiple Choice Questions): Standard single-best-answer items assessing factual knowledge [13].
- CMCQ (Complex Multiple Choice Questions): Multi-statement items requiring simultaneous evaluation and higher-order integrative reasoning [6].
- IBMCQ (Image-Based Multiple Choice Questions): Items containing radiographic images, clinical photographs, or schematic diagrams that require multimodal visual interpretation [11].

### AI models and implementation protocol

Seven consumer AI interfaces were evaluated: GPT-5 (Auto mode), GPT-4o, Gemini 2.5 Pro, Gemini 2.5 Flash, Qwen3-Max, DeepSeek-V3.2-Exp, and Microsoft Copilot. These names reflect the interface labels displayed on the respective consumer platforms during the October–November 2025 evaluation window and were cross-checked against provider nomenclature available for that period. Provider-side version identifiers were unavailable through the consumer interfaces, so the underlying backend models could not be independently verified. GPT-5 (Auto mode) refers to the GPT-5 Auto option displayed in ChatGPT rather than a fixed backend model.

All evaluations were conducted through the official consumer web interfaces using Google Chrome. Evaluation of all seven interfaces was initiated and completed in parallel within the same prespecified October–November 2025 window. Exact day-by-day, session-level dates and timestamps were not retained and could not be reconstructed retrospectively. GPT-5 (Auto mode) and GPT-4o were accessed through a paid ChatGPT subscription, and Gemini 2.5 Pro and Gemini 2.5 Flash through a paid Gemini subscription. Qwen3-Max, DeepSeek-V3.2-Exp, and Microsoft Copilot were accessed through their free consumer tiers. Where platform toggles permitted, web search, memory, and personalization functions were disabled before each session. For interfaces without user-configurable auxiliary settings, these controls were not accessible. Each question was submitted once and only the first response was scored; no repeated-run evaluation was performed. After each response was recorded, the conversation was ended and a new session was opened before the next question, preventing prior-question context from carrying over. Temperature and other decoding parameters were not user-configurable. A zero-shot prompt was used without hints, worked examples, or requests for chain-of-thought reasoning. Accordingly, the findings should be interpreted as the performance of the named consumer interfaces during this evaluation window rather than as estimates for permanently fixed backend model versions. Provider-side changes and the lack of equivalent user-configurable controls across interfaces remain reproducibility limitations.

“Please answer the following question by selecting the correct option: [Question Stem and Options].” For IBMCQs, screenshots were extracted from the official PDF examination booklets without additional processing and uploaded at their original file resolution with the accompanying question text; embedded text was not separately transcribed. The retained study files do not include interface-specific copies of every uploaded image or a prospective image-quality verification log. Consequently, the absence of platform-side resizing or compression and exact pixel-level identity across sessions cannot be independently verified and are acknowledged as reproducibility limitations. The complete standardized prompt and the verifiable elements of the evaluation procedure are reproduced in Appendix S1 (Additional file 1).

Because consumer web interfaces rather than fixed API endpoints were used, provider-side model hashes and backend snapshots were unavailable. GPT-5 (Auto mode) represented an automatically routed interface mode rather than a single identifiable backend model. Consequently, the specific backend model that generated each GPT-5 (Auto mode) response could not be identified. The underlying model used by Microsoft Copilot was likewise not displayed, so response-level backend attribution was also unavailable for that interface. Accordingly, the findings represent the performance of the named consumer interfaces during the evaluation period, not permanently fixed model snapshots.

### Evaluation of responses

Responses were coded as correct or incorrect by comparison with the official DUS answer key. Two expert dentists checked the model-response coding concurrently against the official answer key while using separate computers, and the verified results were entered into a single consolidated dataset. Their role concerned verification of response scoring, not assignment of dental discipline. Because expert-specific ratings were not recorded separately, inter-rater agreement cannot be quantified retrospectively and Cohen’s κ is not reported. Discipline assignment was taken directly from the fixed ÖSYM examination structure and was not independently rated; therefore, inter-rater agreement was not applicable to discipline classification. Question-format labels were assigned using the prespecified structural definitions of MCQ, CMCQ, and IBMCQ.

### Statistical analysis

Data were analyzed using IBM SPSS Statistics v23 and independently recalculated from the question-level dataset using Python. Because every evaluable question was answered by all seven interfaces, comparisons across interfaces were treated as paired. Overall comparisons and exploratory subgroup comparisons stratified by dental discipline, examination period, question format, and discipline-by-format cell were performed using Cochran’s Q test. Only the overall significant omnibus result was followed by 21 exact pairwise McNemar tests with Holm adjustment. No confirmatory pairwise subgroup claims were made because of multiplicity and sparse cells. Cancelled questions were excluded before analysis, and percentages used the evaluable denominator for the corresponding subgroup. Wilson 95% confidence intervals were calculated for overall accuracy. Comparisons of question formats within an individual interface involved different question sets and were descriptive. Because of the small IBMCQ sample, IBMCQ findings were interpreted cautiously. Cramér’s V was not reported for paired comparisons. Two-sided adjusted p values below 0.05 were considered statistically significant.

## Results

**Fig. 1:**
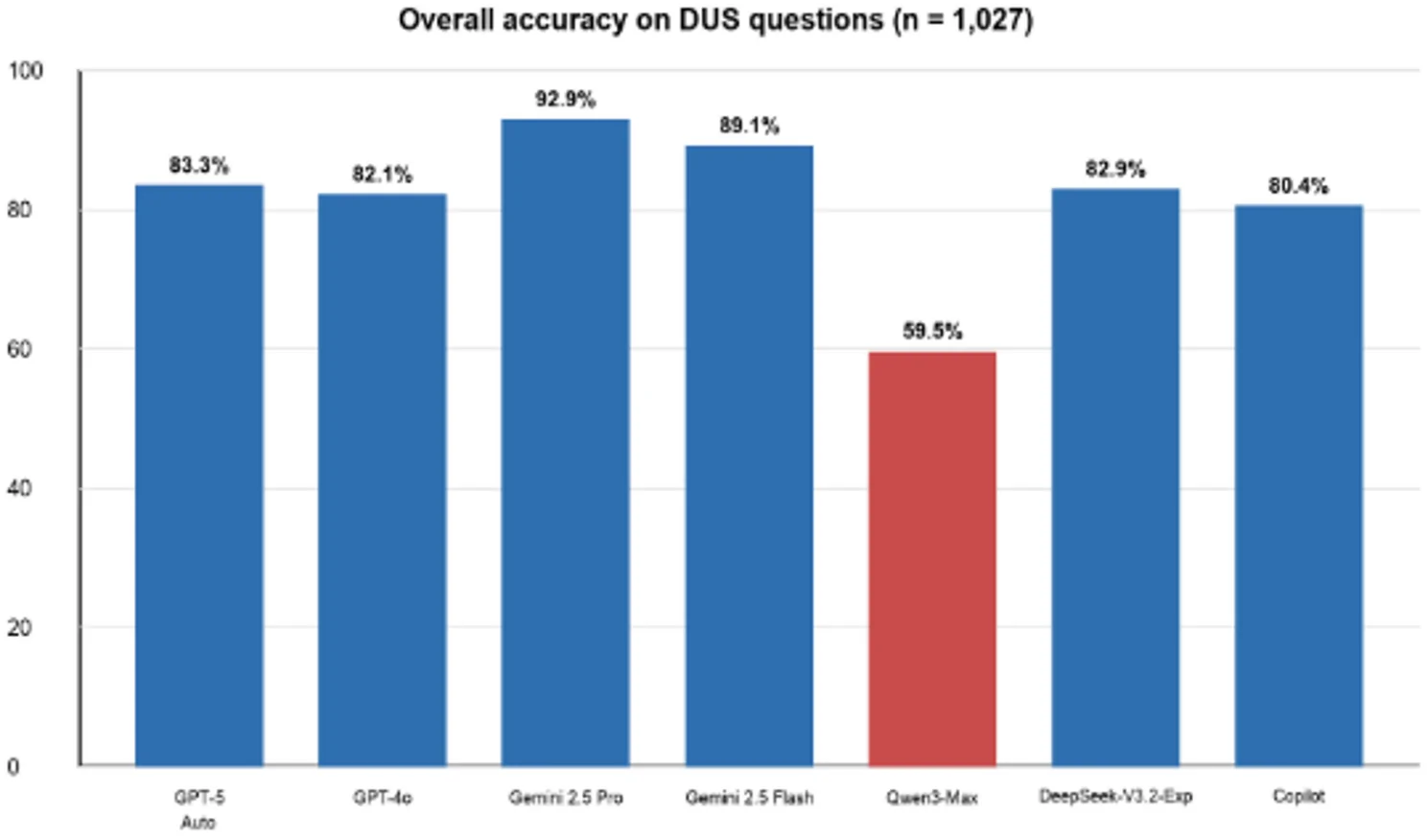
Overall accuracy of seven consumer AI interfaces on evaluable DUS clinical-science questions (2012/1–2021; *n* = 1,027). Error bars indicate Wilson 95% confidence intervals

A total of 1,027 evaluable DUS clinical-science questions from 2012/1 to 2021 were analyzed after exclusion of 13 officially cancelled questions. Overall accuracy differed among the seven interfaces (Cochran’s Q = 669.10; df = 6; *p* < 0.001). Exact pairwise McNemar tests with Holm adjustment identified 15 of 21 model pairs as significantly different. Accuracy was Gemini 2.5 Pro (954/1,027; 92.9%; 95% CI 91.2–94.3%), Gemini 2.5 Flash (915/1,027; 89.1%; 95% CI 87.0–90.9%), GPT-5 (Auto mode) (856/1,027; 83.3%; 95% CI 80.9–85.5%), DeepSeek-V3.2-Exp (851/1,027; 82.9%; 95% CI 80.4–85.0%), GPT-4o (843/1,027; 82.1%; 95% CI 79.6–84.3%), Microsoft Copilot (826/1,027; 80.4%; 95% CI 77.9–82.7%), Qwen3-Max (611/1,027; 59.5%; 95% CI 56.5–62.5%). The six non-significant pairs were GPT-5 (Auto mode) versus GPT-4o; GPT-5 (Auto mode) versus DeepSeek-V3.2-Exp; GPT-5 (Auto mode) versus Microsoft Copilot; GPT-4o versus DeepSeek-V3.2-Exp; GPT-4o versus Microsoft Copilot; DeepSeek-V3.2-Exp versus Microsoft Copilot (Table 1 and Fig. 1).

**Table 1:** Overall accuracy of consumer AI interfaces on evaluable DUS questions (2012/1–2021)

Interface	Correct, *n* (%)	Incorrect, *n* (%)	Wilson 95% CI
Gemini 2.5 Pro	954 (92.9)	73 (7.1)	91.2–94.3
Gemini 2.5 Flash	915 (89.1)	112 (10.9)	87.0–90.9
GPT-5 (Auto mode)	856 (83.3)	171 (16.7)	80.9–85.5
DeepSeek-V3.2-Exp	851 (82.9)	176 (17.1)	80.4–85.0
GPT-4o	843 (82.1)	184 (17.9)	79.6–84.3
Microsoft Copilot	826 (80.4)	201 (19.6)	77.9–82.7
Qwen3-Max	611 (59.5)	416 (40.5)	56.5–62.5

Values are *n* (%) unless otherwise stated. Percentages use *n* = 1,027 evaluable questions. CI, Wilson 95% confidence interval. Overall paired comparison: Cochran’s Q = 669.10, df = 6, *p* < 0.001. Pairwise comparisons used exact McNemar tests with Holm adjustment across 21 pairs; 15 pairs were significant

### Performance across dental disciplines

**Fig. 2:**
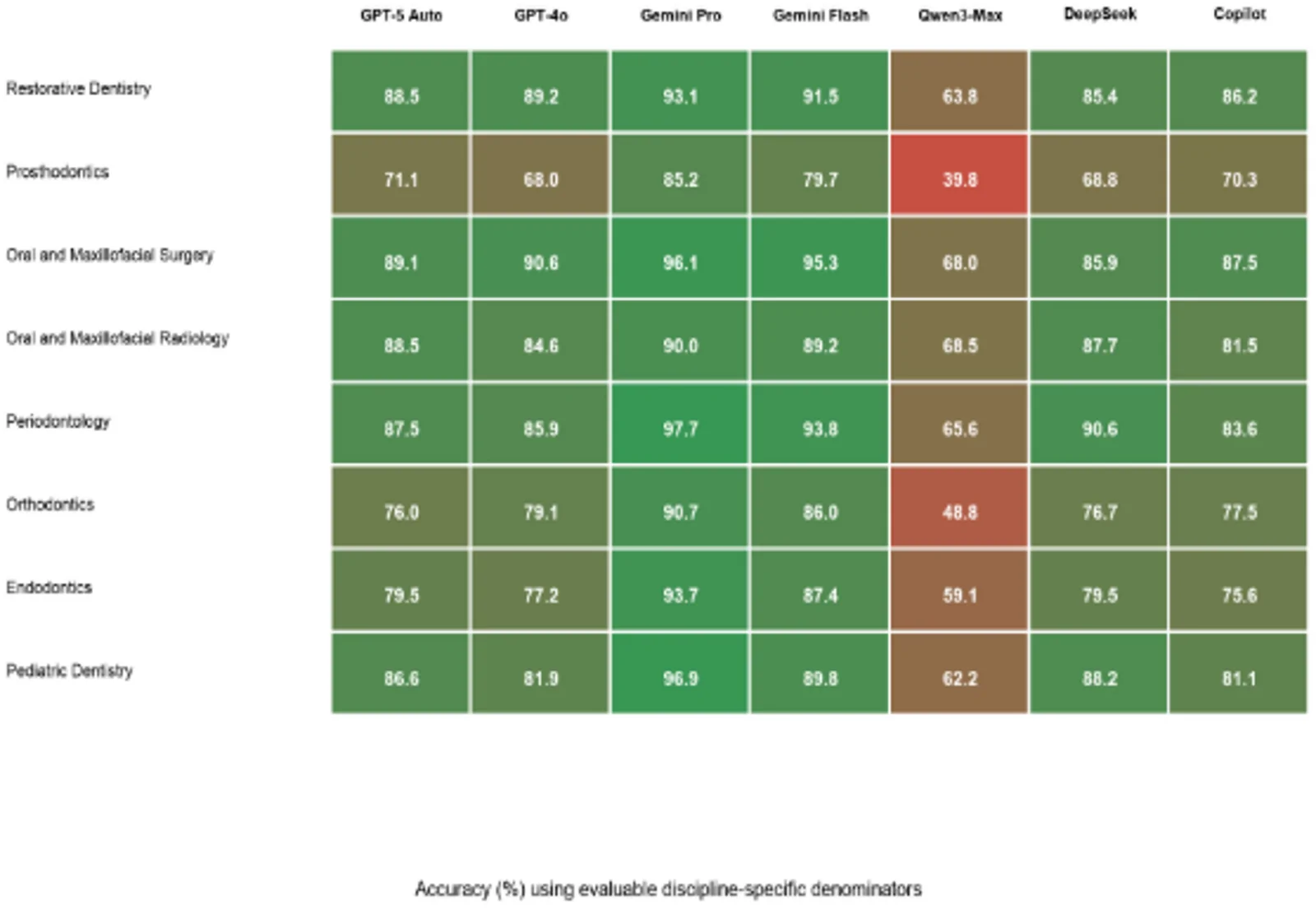
Accuracy (%) by dental discipline and consumer AI interface. Percentages use the evaluable discipline-specific denominator after exclusion of cancelled questions (*n* = 127–130)

Paired subgroup analyses demonstrated differences among interfaces within all eight disciplines (Cochran’s Q, all *p* < 0.001; Table 2). Gemini 2.5 Pro had the highest numerical accuracy in every discipline. Qwen3-Max had the lowest numerical accuracy in every discipline. The magnitude of the difference varied across disciplines, but subgroup rankings should be interpreted cautiously because these analyses were exploratory and involved multiple comparisons (Fig. 2).

**Table 2:** Discipline-specific paired accuracy comparisons of consumer AI interfaces (2012/1–2021)

Discipline	*n*	GPT-5 (Auto mode)	GPT-4o	Gemini 2.5 Pro	Gemini 2.5 Flash	Qwen3-Max	DeepSeek-V3.2-Exp	Microsoft Copilot	Q	*p*
Restorative Dentistry	130	115 (88.5)	116 (89.2)	121 (93.1)	119 (91.5)	83 (63.8)	111 (85.4)	112 (86.2)	85.56	< 0.001
Prosthodontics	128	91 (71.1)	87 (68.0)	109 (85.2)	102 (79.7)	51 (39.8)	88 (68.8)	90 (70.3)	104.97	< 0.001
Oral and Maxillofacial Surgery	128	114 (89.1)	116 (90.6)	123 (96.1)	122 (95.3)	87 (68.0)	110 (85.9)	112 (87.5)	87.84	< 0.001
Oral and Maxillofacial Radiology	130	115 (88.5)	110 (84.6)	117 (90.0)	116 (89.2)	89 (68.5)	114 (87.7)	106 (81.5)	50.90	< 0.001
Periodontology	128	112 (87.5)	110 (85.9)	125 (97.7)	120 (93.8)	84 (65.6)	116 (90.6)	107 (83.6)	96.50	< 0.001
Orthodontics	129	98 (76.0)	102 (79.1)	117 (90.7)	111 (86.0)	63 (48.8)	99 (76.7)	100 (77.5)	106.73	< 0.001
Endodontics	127	101 (79.5)	98 (77.2)	119 (93.7)	111 (87.4)	75 (59.1)	101 (79.5)	96 (75.6)	76.47	< 0.001
Pediatric Dentistry	127	110 (86.6)	104 (81.9)	123 (96.9)	114 (89.8)	79 (62.2)	112 (88.2)	103 (81.1)	91.14	< 0.001

Values are correct responses, *n* (%). The n column gives the evaluable discipline-specific denominator after exclusion of officially cancelled questions. Q and p are from Cochran’s Q test comparing the seven interfaces on the same questions. All subgroup analyses are exploratory

### Performance across examination-year question sets

Paired comparisons across interfaces were significant within every examination-period question set (Cochran’s Q *p* ≤ 0.001; Supplementary Table S2). These period-specific results describe performance on different historical question sets and should not be interpreted as longitudinal changes in the interfaces, because all interfaces were evaluated during the same October–November 2025 period.

Question-format-specific exploratory Cochran’s Q analyses indicated omnibus differences among interfaces for MCQs (*n* = 871; Q = 669.91; *p* < 0.001), CMCQs (*n* = 119; Q = 59.29; *p* < 0.001), and IBMCQs (*n* = 37; Q = 14.68; *p* = 0.023). Gemini 2.5 Pro had the highest numerical accuracy in all three formats: 94.8% for MCQs, 86.6% for CMCQs, and 67.6% for IBMCQs. Because only 37 IBMCQs were available, the omnibus result and percentage estimates are exploratory, have limited reliability, and may be materially affected by the outcome of individual items. No pairwise subgroup tests were performed.

### Discipline-specific performance by question type

**Fig. 3:**
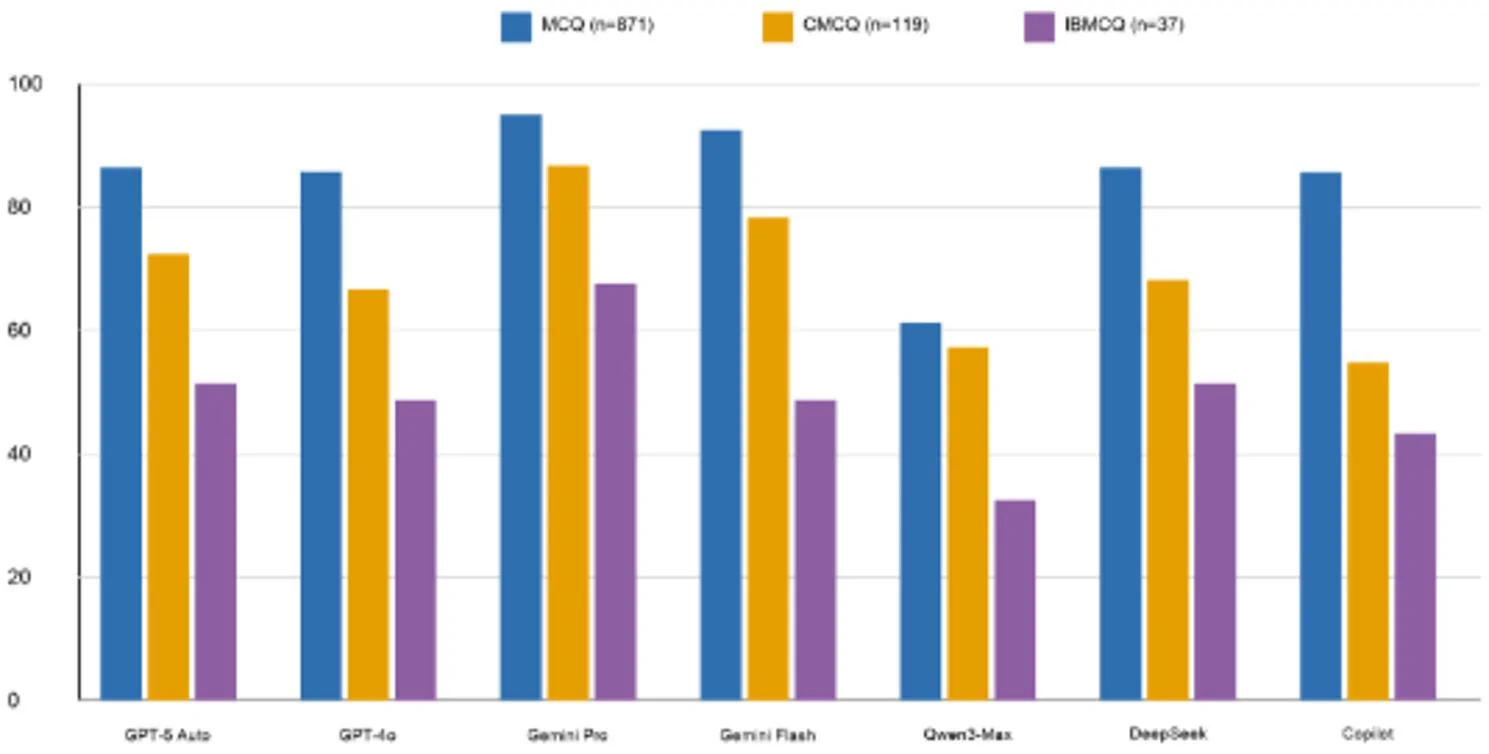
Accuracy by question format for each consumer AI interface. Denominators were 871 MCQs, 119 CMCQs, and 37 IBMCQs

Discipline-by-format results are presented in Table 3 with the exact n for every cell. Exploratory Cochran’s Q tests indicated omnibus differences among interfaces within every MCQ cell (all *p* < 0.001). CMCQ results were more heterogeneous, and IBMCQ cells were frequently too small to support stable inference; several disciplines contained five or fewer IBMCQs, and Periodontology contained only one. Test statistics were reported as not estimable when a cell had insufficient observations or no discordant responses (Fig. 3).

**Table 3:** Discipline-by-question-format accuracy of consumer AI interfaces (2012/1–2021)

Discipline	Format	*n*	GPT-5 (Auto mode)	GPT-4o	Gemini 2.5 Pro	Gemini 2.5 Flash	Qwen3-Max	DeepSeek-V3.2-Exp	Microsoft Copilot	Q	*p*
Restorative Dentistry	MCQ	116	105 (90.5)	105 (90.5)	111 (95.7)	108 (93.1)	78 (67.2)	103 (88.8)	103 (88.8)	75.61	< 0.001
Restorative Dentistry	CMCQ	11	9 (81.8)	10 (90.9)	9 (81.8)	10 (90.9)	5 (45.5)	8 (72.7)	8 (72.7)	10.33	0.111
Restorative Dentistry	IBMCQ	3	1 (33.3)	1 (33.3)	1 (33.3)	1 (33.3)	0 (0.0)	0 (0.0)	1 (33.3)	3.33	0.766
Prosthodontics	MCQ	108	79 (73.1)	74 (68.5)	94 (87.0)	89 (82.4)	42 (38.9)	77 (71.3)	79 (73.1)	104.15	< 0.001
Prosthodontics	CMCQ	18	11 (61.1)	12 (66.7)	14 (77.8)	11 (61.1)	9 (50.0)	10 (55.6)	10 (55.6)	5.51	0.480
Prosthodontics	IBMCQ	2	1 (50.0)	1 (50.0)	1 (50.0)	2 (100.0)	0 (0.0)	1 (50.0)	1 (50.0)	7.00	0.321
Oral and Maxillofacial Surgery	MCQ	111	102 (91.9)	104 (93.7)	108 (97.3)	109 (98.2)	77 (69.4)	100 (90.1)	103 (92.8)	93.86	< 0.001
Oral and Maxillofacial Surgery	CMCQ	12	9 (75.0)	9 (75.0)	12 (100.0)	11 (91.7)	9 (75.0)	9 (75.0)	7 (58.3)	10.00	0.125
Oral and Maxillofacial Surgery	IBMCQ	5	3 (60.0)	3 (60.0)	3 (60.0)	2 (40.0)	1 (20.0)	1 (20.0)	2 (40.0)	5.67	0.462
Oral and Maxillofacial Radiology	MCQ	107	95 (88.8)	92 (86.0)	98 (91.6)	98 (91.6)	71 (66.4)	97 (90.7)	92 (86.0)	63.10	< 0.001
Oral and Maxillofacial Radiology	CMCQ	16	14 (87.5)	13 (81.2)	14 (87.5)	15 (93.8)	14 (87.5)	12 (75.0)	11 (68.8)	6.15	0.406
Oral and Maxillofacial Radiology	IBMCQ	7	6 (85.7)	5 (71.4)	5 (71.4)	3 (42.9)	4 (57.1)	5 (71.4)	3 (42.9)	8.53	0.202
Periodontology	MCQ	113	100 (88.5)	99 (87.6)	110 (97.3)	105 (92.9)	72 (63.7)	102 (90.3)	96 (85.0)	92.88	< 0.001
Periodontology	CMCQ	14	12 (85.7)	11 (78.6)	14 (100.0)	14 (100.0)	12 (85.7)	13 (92.9)	11 (78.6)	10.20	0.116
Periodontology	IBMCQ	1	0 (0.0)	0 (0.0)	1 (100.0)	1 (100.0)	0 (0.0)	1 (100.0)	0 (0.0)	NE	NE
Orthodontics	MCQ	114	91 (79.8)	97 (85.1)	108 (94.7)	104 (91.2)	59 (51.8)	93 (81.6)	94 (82.5)	107.80	< 0.001
Orthodontics	CMCQ	4	3 (75.0)	2 (50.0)	2 (50.0)	2 (50.0)	2 (50.0)	2 (50.0)	1 (25.0)	3.82	0.701
Orthodontics	IBMCQ	11	4 (36.4)	3 (27.3)	7 (63.6)	5 (45.5)	2 (18.2)	4 (36.4)	5 (45.5)	7.71	0.260
Endodontics	MCQ	98	85 (86.7)	85 (86.7)	95 (96.9)	93 (94.9)	66 (67.3)	84 (85.7)	86 (87.8)	58.85	< 0.001
Endodontics	CMCQ	24	14 (58.3)	10 (41.7)	20 (83.3)	15 (62.5)	7 (29.2)	13 (54.2)	9 (37.5)	23.64	< 0.001
Endodontics	IBMCQ	5	2 (40.0)	3 (60.0)	4 (80.0)	3 (60.0)	2 (40.0)	4 (80.0)	1 (20.0)	7.43	0.283
Pediatric Dentistry	MCQ	104	94 (90.4)	90 (86.5)	102 (98.1)	98 (94.2)	66 (63.5)	95 (91.3)	92 (88.5)	98.08	< 0.001
Pediatric Dentistry	CMCQ	20	14 (70.0)	12 (60.0)	18 (90.0)	15 (75.0)	10 (50.0)	14 (70.0)	8 (40.0)	17.54	0.007
Pediatric Dentistry	IBMCQ	3	2 (66.7)	2 (66.7)	3 (100.0)	1 (33.3)	3 (100.0)	3 (100.0)	3 (100.0)	8.67	0.193

Values are correct responses, *n* (%), with the exact denominator shown for every discipline-by-format cell. Q and p are from exploratory Cochran’s Q tests comparing the seven interfaces on the same questions. NE indicates that the paired test was not estimable because of insufficient observations or absence of discordant responses. IBMCQ findings are exploratory because several cells are very small. Superscript-letter displays from the previous version were removed. No pairwise subgroup tests were performed

## Discussion

The paired analysis demonstrated an overall performance hierarchy, with Gemini 2.5 Pro achieving the highest accuracy, followed by Gemini 2.5 Flash, whereas Qwen3-Max had the lowest accuracy. After Holm adjustment, six pairwise comparisons involving GPT-5 (Auto mode), GPT-4o, DeepSeek-V3.2-Exp, and Microsoft Copilot were not statistically significant. These non-significant results do not demonstrate equivalence or comparable performance. The findings show that aggregate performance differed across the evaluated consumer interfaces, but they do not establish stable differences between fixed backend models because some consumer services use automatic routing and provider-side updates. Results attributed to GPT-5 (Auto mode) therefore represent the routed ChatGPT interface, not a single model that can be identified for every response.

Performance also varied across dental disciplines. Gemini 2.5 Pro had the highest numerical accuracy and Qwen3-Max the lowest in every discipline, while Prosthodontics yielded the lowest numerical accuracy for most interfaces. The study was not designed to determine why performance differed across disciplines. These differences therefore should not be attributed to specialty-specific reasoning demands, training data, or model architecture.

The present study extends prior work by evaluating seven interfaces across eight DUS clinical disciplines, three question formats, and 13 examination periods within one standardized Turkish-language dataset. This design allowed discipline- and format-related variation to be examined under a common prompt and scoring framework. Earlier studies using standardized dental multiple-choice questions also reported variation across models, question domains, and evaluation settings [27–29], while broader reviews identified task construction, model generation, multimodal requirements, and educational context as important sources of variation [30–35]. Specialty-specific studies [36–40] and related diagnostic and educational investigations [41–48] provide further evidence that reported performance depends on the domain and evaluation task. These studies support the relevance of the present comparisons but generally examined a narrower range of specialties, formats, or interfaces.

Recent DUS-based studies provide useful context. Gokkurt Yilmaz et al. evaluated eight LLMs using 103 head-and-neck anatomy questions from the 2012–2021 DUS examinations [49]. ChatGPT o1 had the highest overall accuracy, whereas Gemini 2.5 Pro ranked highest in our study. Their topic-based variation parallels the differences we observed among dental disciplines. Yilmaz et al. also found differences between case-based and knowledge-based oral-pathology questions, with ChatGPT o1 leading both groups [50]. We found a similar format effect: CMCQ accuracy was lower than MCQ accuracy for every interface. Our analysis also included IBMCQs and covered eight clinical disciplines. Erdal et al. evaluated the same 2012–2021 DUS prosthodontic question pool under two protocols: independent querying, with a separate chat for each item, and sequential querying in consecutive blocks [51]. Gemini 2.5 Pro showed no significant change between the two protocols, whereas GPT-5 and GPT-4o showed significant declines in knowledge-question accuracy with sequential querying. Our single-attempt, first-response protocol, with a new session for every question, most closely corresponds to their independent-querying condition, although other procedural differences limit direct numerical comparison.

Direct comparison of rankings across studies requires caution because evaluation dates, model generations, interface configurations, routing, prompts, and question composition differed. A key methodological strength of the present study is that all seven interfaces were evaluated on the same question-level dataset with the same standardized prompt and single-response scoring framework. This design reduced confounding from question selection, prompt wording, and response-scoring differences and provided a consistent basis for within-study comparison. However, because exact session settings were not retained in a form that permits retrospective verification, fully identical interface conditions cannot be confirmed. Despite this broader scope, standardized examination performance remains distinct from real clinical decision-making, where history, examination findings, imaging quality, uncertainty, and patient preferences must be integrated. High accuracy on publicly available multiple-choice items therefore does not predict performance in dynamic clinical encounters, where reasoning must extend beyond knowledge retrieval to incorporate patient-specific context, time constraints, and professional judgment.

Question format was associated with marked descriptive differences in accuracy. MCQs produced the highest accuracy for most interfaces, while performance was generally lower for CMCQs and IBMCQs. The exploratory IBMCQ omnibus test was significant, but no pairwise subgroup tests were performed; the small total IBMCQ sample (*n* = 37) and sparse discipline cells preclude strong comparative claims and limit the generalizability of the image-based findings beyond the specific items included in this dataset. This caution is consistent with Yıldırım and Cicek’s IOTN-AC study, in which image-based aesthetic classifications were promising but did not achieve uniformly strong performance across clinical metrics [52]. This comparison reinforces that statistical accuracy on image-based examination items does not establish dependable patient-level assessment.

Clinical-context studies also show that model quality is multidimensional. Cicek and Yıldırım compared eight interfaces on parental questions about early maxillary expansion and found inter-interface differences in accuracy, comprehensiveness, and readability, with no single interface uniformly optimal across all criteria [53]. This supports evaluating consumer AI outputs beyond answer correctness and treating LLMs as supportive rather than replacement tools for expert judgment, particularly when information may influence patient understanding or care.

Pearson’s chi-square and Fisher’s exact tests were not used for between-interface subgroup comparisons because they assume independent observations and do not account for multiple responses obtained from the same questions. Instead, Cochran’s Q treated each question as a matched block and directly accounted for the paired binary responses across the seven interfaces. Subgroup findings were designated as exploratory because numerous omnibus comparisons were performed and several question-format cells were small. No confirmatory pairwise subgroup claims were made.

This study has several limitations. All seven consumer interfaces were evaluated in parallel within the same October–November 2025 window, but exact day-by-day session dates were not retained and could not be reconstructed retrospectively. Web search, memory, and personalization functions were disabled where platform controls permitted. Equivalent controls were not accessible on all interfaces. Provider-side version hashes and backend identities were unavailable. GPT-5 (Auto mode) used automatic routing, preventing identification of the backend model that generated each response, and the model underlying Microsoft Copilot was not exposed. Retained files also did not permit independent verification of pixel-level image identity or platform-side image processing across sessions. Each question was evaluated once, so stochastic variability was not measured. The two experts checked response coding concurrently and recorded the verified results in one consolidated dataset; because expert-specific ratings were not recorded separately, inter-rater agreement could not be quantified and κ was not reported. Turkish-language items may favor interfaces with stronger Turkish capability. Historical DUS questions are publicly available on the ÖSYM website and may have been included in model training data. If so, accuracy may be higher than it would be on unseen questions. The present results cannot distinguish recall of previously encountered material from generalizable reasoning. Subgroup analyses used exploratory paired Cochran’s Q tests; exact McNemar tests with Holm adjustment were restricted to the overall comparison. Examination performance does not establish clinical competence or diagnostic safety. Future validation should use repeated trials and novel, unpublished, clinically contextualized, independently assessed questions.

## Conclusions

In the Turkish DUS context, the evaluated consumer LLM interfaces should not be regarded as interchangeable because their performance varied across interfaces and question formats under standardized conditions. The findings apply to examination-based performance only and do not establish clinical applicability without validation using novel, clinically contextualized, and independently assessed questions.

## Supplementary Information

Supplementary Material 1.

## Data Availability

The datasets supporting the conclusions of this article are included within the article.

## Abbreviations

AI: Artificial Intelligence
LLM: Large Language Model
DUS: Dental Specialization Examination (Diş Hekimliğinde Uzmanlık Eğitimi Giriş Sınavı)
ÖSYM: Assessment, Selection, and Placement Center (Ölçme, Seçme ve Yerleştirme Merkezi)
MCQ: Multiple Choice Question
CMCQ: Complex Multiple Choice Question
IBMCQ: Image-Based Multiple Choice Question
