# Supplementary material for: Accuracy of large language models in the Turkish dental specialization examination (DUS): a multidimensional evaluation across disciplines and question formats
Source: BMC Oral Health. 2026 Sep 1;26:1906. doi: 10.1186/s12903-026-09758-6 (PMC13632068; doi:10.1186/s12903-026-09758-6)
Supplement: Supplementary file 1 [file 12903_2026_9758_MOESM1_ESM.docx]

**Additional file 1**

Supplementary tables for: Accuracy of Large Language Models in the Turkish Dental Specialization Examination (DUS): A Multidimensional Evaluation Across Disciplines and Question Formats

All analyses are restricted to DUS examinations from 2012/1 through 2021. The source dataset contained 1,040 scheduled questions; 13 officially cancelled questions were excluded, leaving 1,027 evaluable questions. Comparisons across AI interfaces use Cochran’s Q test because the same questions were answered by all seven interfaces. Subgroup analyses are exploratory. Superscript-letter displays and independent-samples chi-square tests from the previous supplementary file were removed.

**Supplementary Table S1. Structure of the analytical dataset by discipline and question format.**

| **Discipline** | **MCQ** | **CMCQ** | **IBMCQ** | **Analyzed** | **Cancelled** | **Scheduled** |
| --- | --- | --- | --- | --- | --- | --- |
| Restorative Dentistry | 116 | 11 | 3 | 130 | 0 | 130 |
| Prosthodontics | 108 | 18 | 2 | 128 | 2 | 130 |
| Oral and Maxillofacial Surgery | 111 | 12 | 5 | 128 | 2 | 130 |
| Oral and Maxillofacial Radiology | 107 | 16 | 7 | 130 | 0 | 130 |
| Periodontology | 113 | 14 | 1 | 128 | 2 | 130 |
| Orthodontics | 114 | 4 | 11 | 129 | 1 | 130 |
| Endodontics | 98 | 24 | 5 | 127 | 3 | 130 |
| Pediatric Dentistry | 104 | 20 | 3 | 127 | 3 | 130 |
| Total | 871 | 119 | 37 | 1027 | 13 | 1040 |

Cancelled questions were excluded from every accuracy percentage and inferential test. Source-file spelling variants (IBMCQ, İBMCQ, IBCMCQ, and İBCMCQ) were standardized as IBMCQ.

**Supplementary Table S2. Paired comparison of AI interfaces by DUS examination period.**

| **Period** | **n** | **GPT-5 (Auto mode)** | **GPT-4o** | **Gemini 2.5 Pro** | **Gemini 2.5 Flash** | **Qwen3-Max** | **DeepSeek-V3.2-Exp** | **Microsoft Copilot** | **Q** | **p** |
| --- | --- | --- | --- | --- | --- | --- | --- | --- | --- | --- |
| 2012/1 | 80 | 66 (82.5) | 61 (76.2) | 65 (81.2) | 67 (83.8) | 54 (67.5) | 66 (82.5) | 69 (86.2) | 22.32 | 0.001 |
| 2012/2 | 80 | 66 (82.5) | 66 (82.5) | 75 (93.8) | 75 (93.8) | 55 (68.8) | 67 (83.8) | 68 (85.0) | 36.38 | <0.001 |
| 2013/1 | 78 | 70 (89.7) | 72 (92.3) | 77 (98.7) | 78 (100.0) | 51 (65.4) | 67 (85.9) | 70 (89.7) | 73.57 | <0.001 |
| 2013/2 | 79 | 65 (82.3) | 66 (83.5) | 74 (93.7) | 73 (92.4) | 45 (57.0) | 67 (84.8) | 63 (79.7) | 74.03 | <0.001 |
| 2014/1 | 80 | 71 (88.8) | 67 (83.8) | 75 (93.8) | 72 (90.0) | 41 (51.2) | 66 (82.5) | 65 (81.2) | 95.11 | <0.001 |
| 2014/2 | 79 | 66 (83.5) | 64 (81.0) | 77 (97.5) | 69 (87.3) | 44 (55.7) | 71 (89.9) | 60 (75.9) | 80.79 | <0.001 |
| 2015 | 78 | 67 (85.9) | 68 (87.2) | 73 (93.6) | 73 (93.6) | 36 (46.2) | 66 (84.6) | 65 (83.3) | 112.62 | <0.001 |
| 2016 | 80 | 70 (87.5) | 68 (85.0) | 76 (95.0) | 72 (90.0) | 57 (71.2) | 69 (86.2) | 69 (86.2) | 29.46 | <0.001 |
| 2017 | 75 | 64 (85.3) | 66 (88.0) | 71 (94.7) | 65 (86.7) | 49 (65.3) | 59 (78.7) | 65 (86.7) | 38.27 | <0.001 |
| 2018 | 80 | 58 (72.5) | 59 (73.8) | 68 (85.0) | 59 (73.8) | 36 (45.0) | 61 (76.2) | 50 (62.5) | 62.99 | <0.001 |
| 2019 | 80 | 66 (82.5) | 61 (76.2) | 75 (93.8) | 69 (86.2) | 46 (57.5) | 65 (81.2) | 57 (71.2) | 48.24 | <0.001 |
| 2020 | 80 | 68 (85.0) | 66 (82.5) | 74 (92.5) | 72 (90.0) | 56 (70.0) | 67 (83.8) | 64 (80.0) | 25.68 | <0.001 |
| 2021 | 78 | 59 (75.6) | 59 (75.6) | 74 (94.9) | 71 (91.0) | 41 (52.6) | 60 (76.9) | 61 (78.2) | 65.56 | <0.001 |

Values are correct responses, n (%). Q and p are from exploratory Cochran’s Q tests comparing the seven interfaces on the same questions. These comparisons describe different historical question sets and do not represent longitudinal changes in the interfaces. No confirmatory pairwise subgroup tests were performed.

**Supplementary Table S3. Discipline-specific paired comparisons by DUS examination period.**

Each subtable reports the exact evaluable n for that discipline-period cell. Q and p are from Cochran’s Q test comparing the seven interfaces on the same questions. NE indicates that a test was not estimable because of insufficient observations or no discordant responses. No confirmatory pairwise claims are made for these exploratory cells.

**Supplementary Table S3a. Restorative Dentistry.**

| **Period** | **n** | **GPT-5 (Auto mode)** | **GPT-4o** | **Gemini 2.5 Pro** | **Gemini 2.5 Flash** | **Qwen3-Max** | **DeepSeek-V3.2-Exp** | **Microsoft Copilot** | **Q** | **p** |
| --- | --- | --- | --- | --- | --- | --- | --- | --- | --- | --- |
| 2012/1 | 10 | 7 (70.0) | 7 (70.0) | 6 (60.0) | 6 (60.0) | 7 (70.0) | 6 (60.0) | 8 (80.0) | 4.50 | 0.609 |
| 2012/2 | 10 | 9 (90.0) | 7 (70.0) | 9 (90.0) | 9 (90.0) | 8 (80.0) | 9 (90.0) | 9 (90.0) | 8.67 | 0.193 |
| 2013/1 | 10 | 10 (100.0) | 10 (100.0) | 10 (100.0) | 10 (100.0) | 3 (30.0) | 8 (80.0) | 8 (80.0) | 27.80 | <0.001 |
| 2013/2 | 10 | 10 (100.0) | 10 (100.0) | 10 (100.0) | 10 (100.0) | 6 (60.0) | 8 (80.0) | 7 (70.0) | 14.64 | 0.023 |
| 2014/1 | 10 | 9 (90.0) | 9 (90.0) | 9 (90.0) | 9 (90.0) | 6 (60.0) | 9 (90.0) | 10 (100.0) | 16.50 | 0.011 |
| 2014/2 | 10 | 8 (80.0) | 8 (80.0) | 10 (100.0) | 9 (90.0) | 6 (60.0) | 9 (90.0) | 8 (80.0) | 8.61 | 0.197 |
| 2015 | 10 | 9 (90.0) | 8 (80.0) | 10 (100.0) | 10 (100.0) | 5 (50.0) | 7 (70.0) | 9 (90.0) | 14.57 | 0.024 |
| 2016 | 10 | 8 (80.0) | 8 (80.0) | 10 (100.0) | 8 (80.0) | 7 (70.0) | 9 (90.0) | 9 (90.0) | 7.06 | 0.315 |
| 2017 | 10 | 9 (90.0) | 10 (100.0) | 10 (100.0) | 10 (100.0) | 7 (70.0) | 9 (90.0) | 10 (100.0) | 11.14 | 0.084 |
| 2018 | 10 | 9 (90.0) | 10 (100.0) | 8 (80.0) | 8 (80.0) | 7 (70.0) | 10 (100.0) | 8 (80.0) | 7.71 | 0.260 |
| 2019 | 10 | 9 (90.0) | 10 (100.0) | 10 (100.0) | 10 (100.0) | 6 (60.0) | 10 (100.0) | 8 (80.0) | 14.70 | 0.023 |
| 2020 | 10 | 9 (90.0) | 10 (100.0) | 10 (100.0) | 10 (100.0) | 9 (90.0) | 9 (90.0) | 9 (90.0) | 3.27 | 0.774 |
| 2021 | 10 | 9 (90.0) | 9 (90.0) | 9 (90.0) | 10 (100.0) | 6 (60.0) | 8 (80.0) | 9 (90.0) | 12.00 | 0.062 |

**Supplementary Table S3b. Prosthodontics.**

| **Period** | **n** | **GPT-5 (Auto mode)** | **GPT-4o** | **Gemini 2.5 Pro** | **Gemini 2.5 Flash** | **Qwen3-Max** | **DeepSeek-V3.2-Exp** | **Microsoft Copilot** | **Q** | **p** |
| --- | --- | --- | --- | --- | --- | --- | --- | --- | --- | --- |
| 2012/1 | 10 | 9 (90.0) | 7 (70.0) | 6 (60.0) | 6 (60.0) | 5 (50.0) | 7 (70.0) | 7 (70.0) | 7.07 | 0.314 |
| 2012/2 | 10 | 6 (60.0) | 6 (60.0) | 8 (80.0) | 10 (100.0) | 3 (30.0) | 6 (60.0) | 7 (70.0) | 15.73 | 0.015 |
| 2013/1 | 10 | 7 (70.0) | 9 (90.0) | 10 (100.0) | 10 (100.0) | 6 (60.0) | 9 (90.0) | 9 (90.0) | 11.52 | 0.074 |
| 2013/2 | 10 | 7 (70.0) | 8 (80.0) | 8 (80.0) | 7 (70.0) | 2 (20.0) | 7 (70.0) | 8 (80.0) | 19.20 | 0.004 |
| 2014/1 | 10 | 5 (50.0) | 3 (30.0) | 8 (80.0) | 8 (80.0) | 3 (30.0) | 6 (60.0) | 4 (40.0) | 15.57 | 0.016 |
| 2014/2 | 10 | 7 (70.0) | 6 (60.0) | 9 (90.0) | 8 (80.0) | 5 (50.0) | 9 (90.0) | 6 (60.0) | 12.00 | 0.062 |
| 2015 | 10 | 7 (70.0) | 7 (70.0) | 8 (80.0) | 7 (70.0) | 2 (20.0) | 7 (70.0) | 7 (70.0) | 18.44 | 0.005 |
| 2016 | 10 | 7 (70.0) | 7 (70.0) | 9 (90.0) | 9 (90.0) | 6 (60.0) | 8 (80.0) | 9 (90.0) | 6.00 | 0.423 |
| 2017 | 8 | 5 (62.5) | 5 (62.5) | 6 (75.0) | 5 (62.5) | 5 (62.5) | 3 (37.5) | 6 (75.0) | 3.71 | 0.716 |
| 2018 | 10 | 6 (60.0) | 7 (70.0) | 8 (80.0) | 6 (60.0) | 3 (30.0) | 7 (70.0) | 6 (60.0) | 10.06 | 0.122 |
| 2019 | 10 | 8 (80.0) | 7 (70.0) | 10 (100.0) | 9 (90.0) | 2 (20.0) | 5 (50.0) | 6 (60.0) | 22.80 | <0.001 |
| 2020 | 10 | 8 (80.0) | 8 (80.0) | 9 (90.0) | 8 (80.0) | 7 (70.0) | 7 (70.0) | 7 (70.0) | 2.88 | 0.824 |
| 2021 | 10 | 9 (90.0) | 7 (70.0) | 10 (100.0) | 9 (90.0) | 2 (20.0) | 7 (70.0) | 8 (80.0) | 26.55 | <0.001 |

**Supplementary Table S3c. Oral and Maxillofacial Surgery.**

| **Period** | **n** | **GPT-5 (Auto mode)** | **GPT-4o** | **Gemini 2.5 Pro** | **Gemini 2.5 Flash** | **Qwen3-Max** | **DeepSeek-V3.2-Exp** | **Microsoft Copilot** | **Q** | **p** |
| --- | --- | --- | --- | --- | --- | --- | --- | --- | --- | --- |
| 2012/1 | 10 | 9 (90.0) | 9 (90.0) | 10 (100.0) | 10 (100.0) | 6 (60.0) | 9 (90.0) | 8 (80.0) | 13.33 | 0.038 |
| 2012/2 | 10 | 10 (100.0) | 10 (100.0) | 10 (100.0) | 10 (100.0) | 9 (90.0) | 9 (90.0) | 10 (100.0) | 6.00 | 0.423 |
| 2013/1 | 10 | 10 (100.0) | 10 (100.0) | 10 (100.0) | 10 (100.0) | 10 (100.0) | 10 (100.0) | 10 (100.0) | NE | NE |
| 2013/2 | 10 | 8 (80.0) | 8 (80.0) | 9 (90.0) | 9 (90.0) | 5 (50.0) | 8 (80.0) | 8 (80.0) | 16.29 | 0.012 |
| 2014/1 | 10 | 10 (100.0) | 10 (100.0) | 10 (100.0) | 10 (100.0) | 1 (10.0) | 8 (80.0) | 10 (100.0) | 45.87 | <0.001 |
| 2014/2 | 10 | 9 (90.0) | 9 (90.0) | 10 (100.0) | 10 (100.0) | 7 (70.0) | 10 (100.0) | 8 (80.0) | 11.20 | 0.082 |
| 2015 | 9 | 9 (100.0) | 9 (100.0) | 9 (100.0) | 9 (100.0) | 6 (66.7) | 7 (77.8) | 9 (100.0) | 15.23 | 0.019 |
| 2016 | 10 | 9 (90.0) | 9 (90.0) | 9 (90.0) | 10 (100.0) | 8 (80.0) | 10 (100.0) | 10 (100.0) | 5.14 | 0.526 |
| 2017 | 9 | 8 (88.9) | 8 (88.9) | 8 (88.9) | 7 (77.8) | 6 (66.7) | 7 (77.8) | 7 (77.8) | 3.60 | 0.731 |
| 2018 | 10 | 9 (90.0) | 9 (90.0) | 9 (90.0) | 8 (80.0) | 3 (30.0) | 7 (70.0) | 7 (70.0) | 22.38 | 0.001 |
| 2019 | 10 | 7 (70.0) | 8 (80.0) | 9 (90.0) | 9 (90.0) | 8 (80.0) | 8 (80.0) | 9 (90.0) | 3.60 | 0.731 |
| 2020 | 10 | 9 (90.0) | 9 (90.0) | 10 (100.0) | 10 (100.0) | 10 (100.0) | 9 (90.0) | 9 (90.0) | 3.60 | 0.731 |
| 2021 | 10 | 7 (70.0) | 8 (80.0) | 10 (100.0) | 10 (100.0) | 8 (80.0) | 8 (80.0) | 7 (70.0) | 9.00 | 0.174 |

**Supplementary Table S3d. Oral and Maxillofacial Radiology.**

| **Period** | **n** | **GPT-5 (Auto mode)** | **GPT-4o** | **Gemini 2.5 Pro** | **Gemini 2.5 Flash** | **Qwen3-Max** | **DeepSeek-V3.2-Exp** | **Microsoft Copilot** | **Q** | **p** |
| --- | --- | --- | --- | --- | --- | --- | --- | --- | --- | --- |
| 2012/1 | 10 | 9 (90.0) | 8 (80.0) | 7 (70.0) | 9 (90.0) | 6 (60.0) | 9 (90.0) | 9 (90.0) | 12.40 | 0.054 |
| 2012/2 | 10 | 8 (80.0) | 10 (100.0) | 10 (100.0) | 9 (90.0) | 9 (90.0) | 10 (100.0) | 8 (80.0) | 6.00 | 0.423 |
| 2013/1 | 10 | 8 (80.0) | 8 (80.0) | 9 (90.0) | 10 (100.0) | 7 (70.0) | 10 (100.0) | 9 (90.0) | 8.67 | 0.193 |
| 2013/2 | 10 | 9 (90.0) | 9 (90.0) | 9 (90.0) | 9 (90.0) | 8 (80.0) | 8 (80.0) | 9 (90.0) | 6.00 | 0.423 |
| 2014/1 | 10 | 9 (90.0) | 8 (80.0) | 9 (90.0) | 9 (90.0) | 7 (70.0) | 8 (80.0) | 8 (80.0) | 3.60 | 0.731 |
| 2014/2 | 10 | 9 (90.0) | 9 (90.0) | 10 (100.0) | 9 (90.0) | 8 (80.0) | 10 (100.0) | 8 (80.0) | 6.00 | 0.423 |
| 2015 | 10 | 8 (80.0) | 8 (80.0) | 7 (70.0) | 8 (80.0) | 5 (50.0) | 8 (80.0) | 8 (80.0) | 8.53 | 0.202 |
| 2016 | 10 | 9 (90.0) | 9 (90.0) | 9 (90.0) | 8 (80.0) | 5 (50.0) | 7 (70.0) | 8 (80.0) | 13.50 | 0.036 |
| 2017 | 10 | 9 (90.0) | 9 (90.0) | 10 (100.0) | 9 (90.0) | 8 (80.0) | 9 (90.0) | 8 (80.0) | 3.33 | 0.766 |
| 2018 | 10 | 8 (80.0) | 8 (80.0) | 9 (90.0) | 9 (90.0) | 6 (60.0) | 10 (100.0) | 5 (50.0) | 18.00 | 0.006 |
| 2019 | 10 | 10 (100.0) | 7 (70.0) | 9 (90.0) | 8 (80.0) | 7 (70.0) | 8 (80.0) | 7 (70.0) | 6.00 | 0.423 |
| 2020 | 10 | 9 (90.0) | 8 (80.0) | 9 (90.0) | 10 (100.0) | 5 (50.0) | 9 (90.0) | 9 (90.0) | 12.22 | 0.057 |
| 2021 | 10 | 10 (100.0) | 9 (90.0) | 10 (100.0) | 9 (90.0) | 8 (80.0) | 8 (80.0) | 10 (100.0) | 6.00 | 0.423 |

**Supplementary Table S3e. Periodontology.**

| **Period** | **n** | **GPT-5 (Auto mode)** | **GPT-4o** | **Gemini 2.5 Pro** | **Gemini 2.5 Flash** | **Qwen3-Max** | **DeepSeek-V3.2-Exp** | **Microsoft Copilot** | **Q** | **p** |
| --- | --- | --- | --- | --- | --- | --- | --- | --- | --- | --- |
| 2012/1 | 10 | 9 (90.0) | 8 (80.0) | 9 (90.0) | 9 (90.0) | 7 (70.0) | 9 (90.0) | 9 (90.0) | 5.20 | 0.518 |
| 2012/2 | 10 | 8 (80.0) | 8 (80.0) | 9 (90.0) | 9 (90.0) | 8 (80.0) | 9 (90.0) | 8 (80.0) | 2.25 | 0.895 |
| 2013/1 | 9 | 9 (100.0) | 9 (100.0) | 9 (100.0) | 9 (100.0) | 5 (55.6) | 7 (77.8) | 9 (100.0) | 17.33 | 0.008 |
| 2013/2 | 10 | 8 (80.0) | 8 (80.0) | 9 (90.0) | 9 (90.0) | 8 (80.0) | 10 (100.0) | 9 (90.0) | 8.00 | 0.238 |
| 2014/1 | 10 | 9 (90.0) | 9 (90.0) | 10 (100.0) | 9 (90.0) | 8 (80.0) | 6 (60.0) | 8 (80.0) | 12.75 | 0.047 |
| 2014/2 | 9 | 9 (100.0) | 8 (88.9) | 9 (100.0) | 8 (88.9) | 4 (44.4) | 9 (100.0) | 7 (77.8) | 19.43 | 0.003 |
| 2015 | 10 | 9 (90.0) | 9 (90.0) | 10 (100.0) | 10 (100.0) | 4 (40.0) | 10 (100.0) | 8 (80.0) | 25.30 | <0.001 |
| 2016 | 10 | 9 (90.0) | 8 (80.0) | 10 (100.0) | 10 (100.0) | 7 (70.0) | 9 (90.0) | 8 (80.0) | 10.40 | 0.109 |
| 2017 | 10 | 9 (90.0) | 9 (90.0) | 10 (100.0) | 9 (90.0) | 9 (90.0) | 9 (90.0) | 9 (90.0) | 6.00 | 0.423 |
| 2018 | 10 | 7 (70.0) | 8 (80.0) | 10 (100.0) | 10 (100.0) | 6 (60.0) | 9 (90.0) | 7 (70.0) | 14.18 | 0.028 |
| 2019 | 10 | 9 (90.0) | 9 (90.0) | 10 (100.0) | 10 (100.0) | 10 (100.0) | 10 (100.0) | 9 (90.0) | 4.00 | 0.677 |
| 2020 | 10 | 9 (90.0) | 10 (100.0) | 10 (100.0) | 8 (80.0) | 4 (40.0) | 9 (90.0) | 8 (80.0) | 18.41 | 0.005 |
| 2021 | 10 | 8 (80.0) | 7 (70.0) | 10 (100.0) | 10 (100.0) | 4 (40.0) | 10 (100.0) | 8 (80.0) | 18.94 | 0.004 |

**Supplementary Table S3f. Orthodontics.**

| **Period** | **n** | **GPT-5 (Auto mode)** | **GPT-4o** | **Gemini 2.5 Pro** | **Gemini 2.5 Flash** | **Qwen3-Max** | **DeepSeek-V3.2-Exp** | **Microsoft Copilot** | **Q** | **p** |
| --- | --- | --- | --- | --- | --- | --- | --- | --- | --- | --- |
| 2012/1 | 10 | 6 (60.0) | 5 (50.0) | 8 (80.0) | 8 (80.0) | 6 (60.0) | 7 (70.0) | 9 (90.0) | 10.50 | 0.105 |
| 2012/2 | 10 | 10 (100.0) | 9 (90.0) | 10 (100.0) | 10 (100.0) | 6 (60.0) | 8 (80.0) | 10 (100.0) | 15.47 | 0.017 |
| 2013/1 | 10 | 9 (90.0) | 9 (90.0) | 10 (100.0) | 10 (100.0) | 6 (60.0) | 8 (80.0) | 8 (80.0) | 12.95 | 0.044 |
| 2013/2 | 10 | 7 (70.0) | 8 (80.0) | 10 (100.0) | 10 (100.0) | 4 (40.0) | 8 (80.0) | 8 (80.0) | 15.82 | 0.015 |
| 2014/1 | 10 | 10 (100.0) | 9 (90.0) | 10 (100.0) | 9 (90.0) | 4 (40.0) | 10 (100.0) | 9 (90.0) | 27.43 | <0.001 |
| 2014/2 | 10 | 7 (70.0) | 8 (80.0) | 10 (100.0) | 7 (70.0) | 4 (40.0) | 7 (70.0) | 6 (60.0) | 10.77 | 0.096 |
| 2015 | 10 | 7 (70.0) | 9 (90.0) | 10 (100.0) | 10 (100.0) | 5 (50.0) | 10 (100.0) | 7 (70.0) | 16.97 | 0.009 |
| 2016 | 10 | 9 (90.0) | 10 (100.0) | 10 (100.0) | 10 (100.0) | 7 (70.0) | 9 (90.0) | 8 (80.0) | 9.88 | 0.130 |
| 2017 | 10 | 8 (80.0) | 10 (100.0) | 9 (90.0) | 9 (90.0) | 5 (50.0) | 7 (70.0) | 10 (100.0) | 13.60 | 0.034 |
| 2018 | 10 | 3 (30.0) | 2 (20.0) | 6 (60.0) | 3 (30.0) | 2 (20.0) | 3 (30.0) | 4 (40.0) | 5.85 | 0.440 |
| 2019 | 10 | 8 (80.0) | 8 (80.0) | 8 (80.0) | 9 (90.0) | 3 (30.0) | 7 (70.0) | 6 (60.0) | 16.80 | 0.010 |
| 2020 | 10 | 8 (80.0) | 8 (80.0) | 8 (80.0) | 8 (80.0) | 9 (90.0) | 9 (90.0) | 8 (80.0) | 2.00 | 0.920 |
| 2021 | 9 | 6 (66.7) | 7 (77.8) | 8 (88.9) | 8 (88.9) | 2 (22.2) | 6 (66.7) | 7 (77.8) | 16.69 | 0.011 |

**Supplementary Table S3g. Endodontics.**

| **Period** | **n** | **GPT-5 (Auto mode)** | **GPT-4o** | **Gemini 2.5 Pro** | **Gemini 2.5 Flash** | **Qwen3-Max** | **DeepSeek-V3.2-Exp** | **Microsoft Copilot** | **Q** | **p** |
| --- | --- | --- | --- | --- | --- | --- | --- | --- | --- | --- |
| 2012/1 | 10 | 8 (80.0) | 9 (90.0) | 9 (90.0) | 9 (90.0) | 7 (70.0) | 10 (100.0) | 9 (90.0) | 7.12 | 0.309 |
| 2012/2 | 10 | 7 (70.0) | 8 (80.0) | 9 (90.0) | 8 (80.0) | 8 (80.0) | 8 (80.0) | 8 (80.0) | 1.40 | 0.966 |
| 2013/1 | 10 | 10 (100.0) | 10 (100.0) | 10 (100.0) | 10 (100.0) | 8 (80.0) | 8 (80.0) | 10 (100.0) | 10.91 | 0.091 |
| 2013/2 | 10 | 9 (90.0) | 8 (80.0) | 10 (100.0) | 10 (100.0) | 6 (60.0) | 9 (90.0) | 7 (70.0) | 14.40 | 0.025 |
| 2014/1 | 10 | 9 (90.0) | 9 (90.0) | 9 (90.0) | 9 (90.0) | 6 (60.0) | 9 (90.0) | 8 (80.0) | 13.50 | 0.036 |
| 2014/2 | 10 | 8 (80.0) | 7 (70.0) | 10 (100.0) | 9 (90.0) | 5 (50.0) | 8 (80.0) | 9 (90.0) | 16.80 | 0.010 |
| 2015 | 9 | 8 (88.9) | 9 (100.0) | 9 (100.0) | 9 (100.0) | 5 (55.6) | 9 (100.0) | 9 (100.0) | 18.80 | 0.005 |
| 2016 | 10 | 9 (90.0) | 8 (80.0) | 9 (90.0) | 8 (80.0) | 8 (80.0) | 7 (70.0) | 8 (80.0) | 2.73 | 0.842 |
| 2017 | 8 | 7 (87.5) | 6 (75.0) | 8 (100.0) | 8 (100.0) | 3 (37.5) | 6 (75.0) | 6 (75.0) | 15.25 | 0.018 |
| 2018 | 10 | 6 (60.0) | 6 (60.0) | 9 (90.0) | 6 (60.0) | 3 (30.0) | 7 (70.0) | 5 (50.0) | 14.48 | 0.025 |
| 2019 | 10 | 7 (70.0) | 6 (60.0) | 10 (100.0) | 8 (80.0) | 5 (50.0) | 7 (70.0) | 6 (60.0) | 8.40 | 0.210 |
| 2020 | 10 | 8 (80.0) | 6 (60.0) | 9 (90.0) | 9 (90.0) | 5 (50.0) | 6 (60.0) | 5 (50.0) | 11.00 | 0.088 |
| 2021 | 10 | 5 (50.0) | 6 (60.0) | 8 (80.0) | 8 (80.0) | 6 (60.0) | 7 (70.0) | 6 (60.0) | 4.63 | 0.592 |

**Supplementary Table S3h. Pediatric Dentistry.**

| **Period** | **n** | **GPT-5 (Auto mode)** | **GPT-4o** | **Gemini 2.5 Pro** | **Gemini 2.5 Flash** | **Qwen3-Max** | **DeepSeek-V3.2-Exp** | **Microsoft Copilot** | **Q** | **p** |
| --- | --- | --- | --- | --- | --- | --- | --- | --- | --- | --- |
| 2012/1 | 10 | 9 (90.0) | 8 (80.0) | 10 (100.0) | 10 (100.0) | 10 (100.0) | 9 (90.0) | 10 (100.0) | 7.09 | 0.313 |
| 2012/2 | 10 | 8 (80.0) | 8 (80.0) | 10 (100.0) | 10 (100.0) | 4 (40.0) | 8 (80.0) | 8 (80.0) | 20.16 | 0.003 |
| 2013/1 | 9 | 7 (77.8) | 7 (77.8) | 9 (100.0) | 9 (100.0) | 6 (66.7) | 7 (77.8) | 7 (77.8) | 9.53 | 0.146 |
| 2013/2 | 9 | 7 (77.8) | 7 (77.8) | 9 (100.0) | 9 (100.0) | 6 (66.7) | 9 (100.0) | 7 (77.8) | 9.43 | 0.151 |
| 2014/1 | 10 | 10 (100.0) | 10 (100.0) | 10 (100.0) | 9 (90.0) | 6 (60.0) | 10 (100.0) | 8 (80.0) | 14.70 | 0.023 |
| 2014/2 | 10 | 9 (90.0) | 9 (90.0) | 9 (90.0) | 9 (90.0) | 5 (50.0) | 9 (90.0) | 8 (80.0) | 20.14 | 0.003 |
| 2015 | 10 | 10 (100.0) | 9 (90.0) | 10 (100.0) | 10 (100.0) | 4 (40.0) | 8 (80.0) | 8 (80.0) | 20.79 | 0.002 |
| 2016 | 10 | 10 (100.0) | 9 (90.0) | 10 (100.0) | 9 (90.0) | 9 (90.0) | 10 (100.0) | 9 (90.0) | 4.00 | 0.677 |
| 2017 | 10 | 9 (90.0) | 9 (90.0) | 10 (100.0) | 8 (80.0) | 6 (60.0) | 9 (90.0) | 9 (90.0) | 11.33 | 0.079 |
| 2018 | 10 | 10 (100.0) | 9 (90.0) | 9 (90.0) | 9 (90.0) | 6 (60.0) | 8 (80.0) | 8 (80.0) | 12.00 | 0.062 |
| 2019 | 10 | 8 (80.0) | 6 (60.0) | 9 (90.0) | 6 (60.0) | 5 (50.0) | 10 (100.0) | 6 (60.0) | 10.95 | 0.090 |
| 2020 | 10 | 8 (80.0) | 7 (70.0) | 9 (90.0) | 9 (90.0) | 7 (70.0) | 9 (90.0) | 9 (90.0) | 7.60 | 0.269 |
| 2021 | 9 | 5 (55.6) | 6 (66.7) | 9 (100.0) | 7 (77.8) | 5 (55.6) | 6 (66.7) | 6 (66.7) | 8.28 | 0.219 |

# Appendix S1. Standardized prompt and evaluation procedure

Standardized prompt: “Please answer the following question by selecting the correct option: [Question Stem and Options].”

Each evaluable question was submitted once, in Turkish, to each of the seven consumer AI interfaces, and only the first response was scored. No repeated-run evaluation was performed. After each response was recorded, the conversation was ended and a new session was opened before the next question, preventing prior-question context from carrying over. Evaluation of all seven interfaces was initiated and completed in parallel within the same October–November 2025 window. Exact day-by-day session dates and timestamps were not retained and could not be reconstructed retrospectively. GPT-5 (Auto mode) and GPT-4o were accessed through a paid ChatGPT subscription, and Gemini 2.5 Pro and Gemini 2.5 Flash through a paid Gemini subscription. Qwen3-Max, DeepSeek-V3.2-Exp, and Microsoft Copilot were accessed through their free consumer tiers. Where platform toggles permitted, web search, memory, and personalization functions were disabled before each session. Equivalent controls were not accessible on all interfaces. Temperature and other decoding parameters were not user-configurable. The prompt contained no hints, worked examples, or requests for chain-of-thought reasoning.

For IBMCQs, screenshots extracted from the official examination booklets were uploaded with the question text. Embedded image text was not separately transcribed. Interface-specific image copies and a prospective image-quality verification log were not retained; therefore, pixel-level identity and platform-side resizing or compression across sessions cannot be independently verified. The first response was compared directly with the official ÖSYM answer key.
